# Endoparasites of Domesticated Animals That Originated in the Neo-Tropics (New World Tropics)

**DOI:** 10.3390/vetsci6010024

**Published:** 2019-03-06

**Authors:** Kegan Romelle Jones, Gary Wayne Garcia

**Affiliations:** 1 The Department of Basic Veterinary Sciences (DBVS), School of Veterinary Medicine (SVM), Faculty of Medical Sciences (FMS), The University of the West Indies (UWI), Mt. Hope, Trinidad and Tobago; 2 The Open Tropical Forage-Animal Production Laboratory (OTF-APL), Department of Food Production (DFP), Faculty of Food and Agriculture (FFA), The University of the West Indies (UWI), St. Augustine, Trinidad and Tobago; prof.gary.garcia@gmail.com

**Keywords:** *Cavia porcellus*, *Meleagris gallopavo*, *Chinchilla lanigera*, South American camelids, *Carina moschata*, *Anas platyrhynchos*, *Dendrocyga autumnalis*

## Abstract

This review serves to summarize parasites found in Domesticated animals which were found in the Neo-Tropics. Indigenous domesticated Neo-tropical animals include South American camelids, (*Lama gunacoa*, *Lama glama*, *Lama pacos*, *Vicuna vicuna*), guinea pigs (*Cavia porcellus*), chinchillas (*Chinchilla lanigera*), turkeys (*Meleagris gallopavo*) and ducks (*Cairina moschata*, *Anas platyrhynchos*, *Dendrocyga autumnalis*). These animals were chosen due to their origin of existence (Neo-tropics) and over time these animals became domesticated and were distributed throughout the world. Over eighty (80) references were collected for this review and the papers spanned over eighty (80) years from 1934 to 2018. The gastrointestinal parasites reported for each animal were tabulated and their effects in the animal noted. Parasites reported in domesticated Neo-tropical animals had little to no effect on wild and free ranging animals with a few cases of illness and decreased productivity. The majority of articles viewed these animals as reservoir host which can infect humans and other domesticated livestock. It must also be noted that research done in the past did not focus on the effect these parasites had on these animals but only observed their potential as reservoirs for parasitic diseases.

## 1. Introduction

Livestock which are present in the Neo-tropics can be divided into three categories. The first group was domesticated animals introduced in to the Neo-tropics. These included cattle (*Bos indicus*, *Bos taurus*), sheep (*Ovis aries*), goat (*Capri hircus*), chickens (*Gallus domesticus*), horses (*Equus caballus*) and pigs (*Sus sucrofa*). The second group was domesticated animals which were found in the Neo-tropics. These included South American Camelids (SAC) (*Lama gunacoa*, *Lama glama*, *Lama pacos Vicuna vicuna*), guinea pigs (*Cavia porcellus*), chinchillas (*Chinchilla lanigera*), turkeys (*Meleagris gallopavo*) and ducks (*Cairina moschata*, *Anas platyrhynchos*). These animals have been used by humans for many purposes. South American camelids have been used for fur and meat, guinea pigs have been used for meat and laboratory research, chinchillas have been used in the production of fur coats, turkeys and ducks have been used for meat production [1]. The third groups of animals are Non-domesticated Neo-tropical animals. These included the agouti (*Dasyprocta leporina*), lappe (*Agouti paca*), manicou, (*Didelphis marsupialis insularis*) red brocket deer (*Mazama americana*), capybara (*Hydrochaerus hydrochaeris*) and collared peccary (*Peccari tajucu*) [2].

Little information is known on the effect of these parasites on the non-domesticated neo-tropical animals. In recent times some work has been done on the reproductive system of the agouti [3,4], nutrition of the agouti [5,6,7], parasites in wild and captive reared agoutis [8,9,10], the blood profile of agoutis reared in captivity [11], biology of the manicou [12] and digestive anatomy of the red brocket deer [13].

Parasites which were found in the domesticated species introduced into the Neo-tropics have been well documented [14,15,16]. Recently, the gastrointestinal parasites present in these animals were reviewed. Parasites were summarized as organisms which negatively affected the animals’ health and performance by removal of essential nutrients and its effect can be seen clinically or sub-clinically [17].

The objectives of this review were (1) to identify the gastrointestinal parasites of South American Camelid (SAC) (*Lama gunacoa*, *Lama glama*, *Lama pacos Vicuna vicuna*), guinea pig (*Cavia porcellus*), chinchilla (*Chinchilla lanigera*), turkey (*Meleagris gallopavo*) and duck (*Cairina moschata*, *Anas platyrhynchos*). These domesticated Neo-tropical animals have been reported in different locations. (2) To record the effects that these parasites have been reported to have had on the health and production of these animals.

## 2. Materials and Method

For the purpose of this review, reports and articles were searched for in scholarly publication databases (Google Scholar, PubMed and UWI linc). Search terms used were specific species names (e.g., *Lama gunacoa*, *Lama glama*, *Lama pacos Vicuna vicuna*, guinea pig (*Cavia porcellus*), chinchilla (*Chinchilla lanigera*), turkey (*Meleagris gallopavo*) and duck (*Cairina moschata*, *Anas platyrhynchos*) combined with the term endoparasites. Additional key terms such as “South American camelids” and “domesticated animal parasites” were also used. Searches were conducted from earliest available date in the database up to December 2018 and any other information was not presented in the review. All sources were assessed by the author for relevance, credibility and scientific inclusion to ensure the thoroughness and accuracy of review. Papers which focused on blood parasites or ectoparasites were not included in this review. Over 120 articles were retrieved but sixty-six articles were chosen for the body of this review based on relevance. The Section 3, Section 4, Section 5, Section 6 and Section 7 give information on the relevant Neo-tropical species. The section on South American Camelids was searched and twenty-five (25) articles were identified in the search. Of these twenty-five (25) papers only eleven (11) were relevant to endoparasites in these animals. The section on guinea pigs was searched using databases mentioned above and twenty (20) articles were identified in the search. Of these twenty articles (20) only thirteen (13) were relevant to endoparasites in these animals. The section on chinchillas was searched using databases mentioned above and fifteen (15) articles were identified in the search. Of these fifteen (15) papers only seven (7) were relevant to endoparasites in these animals. The section on turkeys was searched and thirty (30) articles were identified in the search. Of these thirty (30) papers only fifteen (15) were relevant to endoparasites in these animals. The final section which was done on turkeys was searched and thirty (30) articles were identified in the search. Of these thirty (30) papers only eighteen (18) were relevant to endoparasites in these animals.

## 3. South American Camelids (*Lama gunacoa*, *Lama glama*, *Lama pacos*, *Vicuna vicuna*)

Digestive tracts of Camelids in South America were analysed and *Graphinema auchenia*, *Nematodirus lamae* and *Spiculopteragia peruviana* was observed [18]. The following parasites were also identified; *Camelostrongylus* spp., *Ostertagia* spp., *Teladorsagia* spp., *Marshallagia* spp., *Haemonchus* spp., *Trichostrongylus* spp., *Nematodirus* spp., *Capillaria* spp., *Cooperia* spp., *Bunostomum* spp., *Chabertia* spp., *Lamanema* spp., *Strongyloides* spp., *Oesophogostamum* spp., *Trichuris* spp. and *Skrjabinema* spp. Trematodes included *Fasciola hepatica*, *Fascioloides magna* and *Dicrocoelium dendriticum. Moniezia* spp. was the only cestode mentioned. Intestinal protozoa found were *Cryptosporidium* spp., *Gardia* spp. and *Eimeria* spp. [18,19]. Lamas were classified as New World Camelid (NWC), at present they were distributed throughout the world but these animals originated in South America. Parasitic organisms found in these animals cause severe clinical disease. In England and Wales parasitic gastroenteritis (PGE), fascioliasis, coccidiosis and cryptosporidiosis were the major causes of gastrointestinal health problems [20]. *Bunostomum*, *Camelostrongylus*, *Capillaria*, *Trichostrongylus*, *Cooperia*, *Nematodirus*, *Ostertagia*, *Marshallagia* and *Trichuris* spp. was found in the stomach and intestines of NWC in England and Wales. Pathological lesions such as inflammation of the 3rd compartment of the stomach (acid pepsin stomach), enlarged mesenteric lymph nodes, subcutaneous oedema, ascites, hydrothorax and pulmonary oedema were found in lamas which had the gastrointestinal protozoa and helminths [20]. In Wisconsin a case of a young lama with unformed stool and poor condition was reported. Faecal examination revealed a few strongyles, *Nematodirus* spp. and *Giardia* cysts [21].

In Britain, two clinical cases of llamas (*Lama glama*) aged four (4) month and eight (8) were reported to have had diarrhoea, weight loss and subsequently died. Faecal examination found *Camelostrongylus mentalatus* and *Trichostrongylus* spp. in the third compartment of the stomach. In the large intestines *Nematodirus*—like eggs, *Trichuris*—like worms, *Eimeria macusaniensis* and *Eimeria* spp. [22]. In Chile, *Nematodirus* spp., *Eimeria macusaniensis* and Strongylida—like eggs were found in the faeces of llamas reared in semi-captivity. The animals’ health or body condition was not reported in this study [23] (Table 1). Neonatal diarrheal in lamas and alpacas was reviewed and parasitic causes were found to be due to *Cryptosporidium* spp., *Giardia duodenalis* and *Eimeria* spp. [24]. In the southern United States from 2002–2013 lamas and alpacas were analysed for *Haemonchus contortus*. There was a prevalence of 42.6% in lamas and 22.2% in alpacas. There was a correlation between faecal egg count (FEC) and haematocrit, haemoglobin and red blood cells. Gross lesions seen included peritoneal, thoracic and pericardial effusions. Visceral pallor, subcutaneous oedema and serous atrophy of fat were also found [25]. South American Camlids were found to be infected with numerous *Eimeria* spp. Authors found the five most pathogenic species to be *Emeria macusaniensis*, *Eimeria lamae*, *Eimeria alpacae*, *Eimeria punoensis* and *Eimeria ivataensis*. With *Eimeria macusaniensis* considered as the most pathogenic species for South American Camelids [26].

The use of fenbendazole as an anthelmintic showed to have no adverse effects on the llamas’ health at 5 mg/kg. Reduction of faecal egg load was observed for *Strongyloides*, *Trichuris* and *Nematodirus* as compared to the control group [27]. Lamas and alpacas in Belgium were treated with ivermectin and showed no sign of clinical illness at 0.2 mg/kg. The results showed that there was a 100% reduction in faecal egg count for *Oesophagostomum* spp. and *Trichostrongylus* spp. [28]. Reduction of coccidiosis can be attained through good management practices and maintenance of hygienic facilities. Strategic use of anticoccidials on a heard basis can be done. Anticoccidials used included decoquinate at 0.5 mg/kg/day for 28 days, amprolium at 5 mg/kg/day for 21 days. Ionophore antibiotics such as salinomycin and monensin should not be used as they were reported to be toxic in camels [19,24] (Table 2). The effect of parasitic treatment on the performance of weaning alpaca was investigated. It was noted that animals which were dewormed performed worse than animals that were not dewormed. This information was preliminary but is does shed light on the role these organisms may play in the digestive tract of the animal [29].

## 4. Guinea Pigs (*Cavia porcellus*)

Caecal flagellates were found in the normal fauna of the guinea pig and treatment to remove these organisms may not have an effect on the animal. These caecal flagellates included *Caviomonas mobilis*, *Chilomastix intestinalis*, *Chilomastix wenrichi*, *Chilomitus caviae*, *Chilomitus conexus*, *Enteromonas caviae*, *Hexamastix caviae*, *Hexamastix robustus* and *Tritrichomonas caviae* [33]. Protozoa in the gastrointestinal tract which were reported to have caused disease included: *Giardia duodenalis*, *Cryptosporidium wrairi*, *Eimeria caviae* and *Balantidium caviae*. Helminths reported to have caused clinical disease included *Fasciola* spp. and *Paraspidodera uncinata*. Clinical disease included weight loss, debilitation and diarrhoea [33]. Helminths which were reported to be non-clinical included *Trichuris gracilis*, *Capillaria hepatica*, *Viannella travassosi*, *Graphidiodes mazzai*, *Pseudoquinqueserialis caviae*, *Taxorchis caviae*, *Taxorchis ringueleti*, *Anoplocephala* spp. and *Monoecocestus parcitesticulatus* [33].

In Peru, wild guinea pigs were examined in three localities in the Andean Highlands. *Capillaria hepatica*, *Graphidoides mazzai*, *Trichuris gracilis*, *Paraspidodera uncinata* and *Fasciola hepatica* was found in the gastrointestinal tract however no reference to the animals’ body condition or body weight was recorded [34]. In Brazil worm burdens were compared in guinea pigs conventionally and openly reared. Guinea pigs in the study ranged from 240g to 1000 g. Animals reared conventionally had 10% prevalence of *Paraspidodera uncinata* while those reared openly had 40% prevalence [35].

In Italy, pet guinea pigs were analysed for endoparasites using faecal samples. Intestinal parasites found included *Nippostrongylus*-like eggs, *Paraspidodera uncinata* and *Eimeria caviae* [36]. In Italy faeces of pet rodents were sampled for *Hymenolepis* spp. The survey sampled sixty (60) guinea pigs and all were free of *Hymenolepis* spp. using floatation techniques [37]. Laboratory guinea pigs which were housed with rabbits were randomly tested for gastrointestinal parasites. Guinea pigs had *Paraspidodera uncinata* and *Balantidium coli.* Guinea pigs used in this study had similar weights (600g) but no record of the body condition or clinical history of the animals was given [38]. Faecal samples of pet guinea pigs were analysed using polymerase chain reaction (PCR) in Europe for molecular typing of *Giardia* spp. Upon analysis 4% of the animals sampled were positive for *Giardia* spp. [39].

In the Congo, guinea pigs are farmed as a source of meat for rural villages. Mortalities were found to be highest for the first week of the guinea pig’s life and more than half of the deaths were due to parasitic diseases. The two gastrointestinal parasites that were found to be the cause of the mortality in young guinea pigs were *Balantidium coli* and *Eimeria* spp. [40]. In the Highlands of Cameroon farmed guinea pigs and guinea pigs housed at teaching centres were tested for endoparasites. The study found *Graphydium strigosum*, *Trichostrongylus* spp. and *Paraspidodera uncinata* in guinea pigs reared on farm. At the teaching centres *Eimeria caviae* and *Paraspidodera uncinata* were identified using faecal floatation techniques. However, this study failed to record the body weights or body condition of the animals’ sampled [41] (Table 3).

In rural villages in Bolivia small scale mixed farming systems are used as the main source of meat protein for rural villages. The major concern reported was that of theft and were reported to be problem free [42]. In these villages the Guinea-pig provides meat and a flexible source of income. Mortality in this study was attributed to inadequate nutrition [43]. *Eimeria caviae*, *Cryptosporidium wraira* and *Balantidium coli* was reported to be found in guinea pigs and have low pathogenic effect. *Paraspidodera uncinata* has been reported to be found in guinea pigs but treatment included use of piperazine [31]. *Eimeria caviae* has been reported to cause haemorrhagic diarrhoea in South America. Recommended treatment for coccidian in guinea pigs included to the use of sulphaquinalones [30] (Table 2). In Australia, guinea pigs were necropsied and lung and intestinal tissue were analysed. Using faecal floatation techniques *Cryptosporium* was identified in 24% of the animal samples. Molecular analysis using Polymerase Chain Reaction (PCR) was used and *C. homai* was the species identified [44].

## 5. Chinchillas (*Chinchilla lanigera*)

In Europe, faecal samples of chinchillas were collected by veterinarians. The samples were positive for *Giardia* Assemblage A and B which causes clinical illness in humans [39]. In Italy rodents were surveyed for gastrointestinal parasites and *Hymenolepis* spp. was found in 25% of chinchillas sampled [36]. Chinchillas reared in Italian breeding facilities were sampled for *Giardia duodenalis* infection. The results showed *Giardia duodenalis* assemblage B and C was present using molecular techniques. The survey was conducted to evaluate the risk of zoonotic diseases but the health of the animals or body condition of the animals was not recorded [45]. In China faecal samples from pet chinchillas were analysed. Genotyping of *Giardia duodenalis* found assemblage A and B using molecular techniques [46].

In Chile, *Giardia* spp. was found in chinchillas of both commercial breeding facilities and natural reserve. The animals held at the reserve had a higher prevalence of *Giardia* spp. as compared to the commercial breeding farm. The faecal samples that were analysed were not given with prior clinical history or body condition of the animals [47]. In Brazil captive chinchillas were sampled for gastrointestinal parasites. These animals had no previous history of gastrointestinal illness but only *Giardia* spp. was identified in 8% of the samples [48].

In Belgium, pet chinchillas and breeding animals were sampled for the presence of *Giardia duodenalis*. Faecal samples were analysed using faecal floatation and molecular techniques but the animals’ clinical history or body condition were not taken. The study found 66.3% positive for *Giardia duodenalis* with the predominant assemblage being B with assemblages C, A and E also present [49]. A similar study was done in Brazil with respect to the prevalence of *Giardia duodenalis* from exotic and wild animals kept in captivity. Chinchillas were found to be positive for *Giardia duodenalis* assemblage B which is zoonotic. All animals were asymptomatic for gastrointestinal illness [50]. In Argentina, in a fourteen-year study on fatal illness of captive chinchillas were performed. Gastrointestinal parasites found were *Giardia* spp., *Eimeria* spp., *Trichostrongylus* spp., *Trichuris* spp., *Cryptosporidium* spp. and *Saccharomyces glutauratus*. The authors noted that these parasites were not present in sufficient number to have caused death or illness [51] (Table 4). A similar study was done in Brazil but lasted for four years. Two hundred and two cases were examined and only one case of *Giardia* infection was attributed to death [52].

## 6. Turkeys (*Meleagris gallopavo*)

In Philadelphia, gastrointestinal samples were taken from wild turkeys and three cestodes were identified. There were *Davainea ransomi*, *Davainea fuhrinanni* and *Metroliasthes lucida* [53]. In Pennsylvania, wild turkeys (*Melegradis gallopavo silvestris* Vieollot) were surveyed for protozoan parasites. The species of gastrointestinal protozoa encountered were *Eimeria meleagridis*, *Histomonas meleagridis* and *Trichomonas* spp. No information on the body condition or live weight of the wild turkeys sampled were taken, therefore the effect of these protozoan parasites on wild turkey populations were unknown [54].

In Florida, wild turkeys were examined for helminths at different locations. The study revealed ten (10) trematodes, six (6) cestodes, seventeen (17) nematodes and one (1) acanthocephalan. Trematodes identified were: *Echinoparyphium recurvatum*, *Zygocotyle lunata*, *Stomylotrema vivarium*, *Strigea elagans meleagris*, *Prostogonimus ovatus*, *Echinostoma revolutum*, *Ascocotyle* spp., *Brachylaima virginianum*, *Tanaisia* spp. and *Zonorchis* spp. [55]. Cestodes found were: *Metroliasthes lucida*, *Raillietina geogiensis*, *Raillietina ransomi*, *Raillietina cesticillus*, *Davainea meleagridis* and *Hymenolepsis carioca* [54]. Nematodes recorded were: *Strongyloides* spp., *Trichostrongylus tenuis*, *Dispharynx nasuta*, *Cyrnea eurycerca*, *Ascaridia dissimilis*, *Capillaria* spp., *Cyrnea* spp., *Singhfilaria hayesi*, *Heterakis gallinarum*, *Aproctella stoddardi*, *Synlimantus* spp., *Aulonocephalus pennula*, *Capillaria* spp., *Chandlerella* spp., *Cheilospirura spinose*, *Splendidofliaria* spp. and Slendidofilariinae [55]. The one acanthocephalan observed was *Mediohynchus papillosum*. The authors did not record the live-weight or body condition of the wild turkeys to investigate the effect of the parasites on the wild turkeys [56].

In southern United States, wild turkeys (*Meleagradis gallopovo*) were analysed for the presence of gastrointestinal helminths. The study found four species of trematodes which were: *Brachylaema virginiana*, *Cotylurus flabelliformis*, *Echinoparyphium recurvatum* and *Zygocotyle lunata*. Eight species of cestodes were identified: *Davainea meleagradis*, *Hymenolepis cantaniana*, *Hymenolepis carioca*, *Hymenolepis* spp., *Metroliasthes lucida*, *Raillietina georgiensis*, *Raillietina williamsi* and *Raillietina ransomi*. [53] Thirteen species of nematodes were identified: *Ascaridia dissimilis*, *Ascaridia galli*, *Capillaria annulata*, *Capillaria bursata*, *Capillaria obsignata*, *Dispharynx nasuta*, *Gongylonema ingluvicola*, *Heterakis gallinae*, *Seurocyrnea colini*, *Seurocyrnea* spp., *Strongyloides avium*, *Strongyloides* spp. and *Trichostrongylus tenuis*. However, no records were presented on the body condition or body weight of the wild turkeys that were sampled [56]. In Kentucky and Tennessee gastrointestinal tracts were taken from wild turkeys. Parasites collected from these wild birds were *Hymenolepis carioca*, *Metroliasthes lucida*, *Raillietina georgensis*, *Raillietina williamsi*, *Ascaridia dissimilis*, *Capillaria caudinflata* and *Heterakis gallinarum*. All animals’ samples that were collected did not include the animals’ body condition or health status [57].

Pen-raised wild turkeys were surveyed for coccidian parasites, gastrointestinal samples were taken but no information was recorded on the weight, body condition or clinical history of the sampled birds. The observations revealed *Eimeria meleagrimitis*, *Eimeria gallopavonis*, *Eimeria meleagridis*, *Eimeria dispersa*, *Eimeria innocua-Eimeria subrontunda* and an undescribed coccidian species [58]. Wild turkeys in Arkansas were sampled for infectious disease organisms. Neither the live-weight nor body condition of turkeys sampled were recorded. Enteric parasites found were *Ascaridia dissimilis*, *Heterakis gallinarum*, *Eimeria dispersa* and *Raillietina* spp. [59]. Juvenile and adult turkeys in eastern Kansas were sampled for enteric helminths. The animals which were collected were eviscerated and the contents stored in formalin. However, the body weight or body condition was not recorded. The parasites that were found included; *Echinoparyphium recurvatum*, *Echinostoma revolutum*, *Metroliasthes lucida*, *Imparmargo baileyi*, *Railletiena* spp., *Choanataenia* spp., *Mediorhynchus grandis* and *Heterakis gallinarum* [60].

In Brazil, free range turkeys were sampled for gastrointestinal parasites. The birds sampled showed no clinical sign of disease and had weights ranging from 2.2 kg to 8.87 kg. Helminths found were *Cheilospirura hamulosa*, *Heterakis gallinarum*, *Baruscapillaria obsignata*, *Eucoleus annulatus*, *Hymenolepis cantaniana* and *Raillietina tetragona*. Microscopic lesions of the gastrointestinal tract included intense chronic diffuse inflammation mixed granulocyte inflammation extended to the muscular layer [61,62,63,64].

In Nebraska, wild turkeys were surveyed for infectious disease. Samples taken were visceral organ but body condition or live-weight was not recorded. Cestodes found included: *Choanotaenia infundibulum*, *Davainea meleagridis*, *Echinolepsis carioca*, *Fimbriaria fasciolaris*, *Microsomacanthus paracompressa*, *Raillietina circumvallata*, *Raillietina cestillus*, *Raillietina echinoborthida*, *Raillietina ransomi*, *Raillietina tetragona*, *Raillietina williamsi* and *Sobolevicanthus gracilis* [64]. Nematodes identified were *Ascaridia dissimilis*, *Ascaridia galli*, *Capillaria bursata*, *Capillaria caudinflata*, *Capillaria annulatus*, *Eucoleus annulatus*, *Ganguleterakis dispar*, *Heterakis gallinarum*, *Syngamus trachea*, *Thominx phasiania* and *Trichostrongylus tenuis* [65]. Trematodes recorded were *Echinoparyphium recurvatum*, *Echinoparypium rubrum*, *Echinostoma revolutum*, *Echinostoma trivolis*, *Notocotylus attenuatus*, *Prosthogonimus cuneatus* and *Prostogonimus ovatus*. The one acanthocephalan observed was *Mediorynchus* spp. [65].

Turkey poults from farms experiencing diarrhoea, depression and huddling were sampled. The causative agent was *Cryptosporidium* spp. Present in the enterocytes of the middle and lower small intestine. Affected poults showed villous atrophy, crypts that were hypertrophied and reduction in total mucosal thickness [66]. In Iowa commercial turkeys were sampled and *Cryptosporidium* oocyst was found in 80% of seventeen day old turkeys and 38% in twenty-four day old turkeys. Birds were not clinically ill from the pathogen and no histological lesions were observed except for displacement of micro-villi at attachment sites [67] (Table 5). Wild male turkeys were infected with coccidia to observe the effect on plumage coloration. Turkeys were infected with *Eimeria adenoids*, *Eimeria gallogavonis*, *Eimeria meleagramitis* and *Eimeria dispersa*. Results showed turkeys infected with coccidia had plumage coloration that was duller than un-infected group [68].

## 7. Ducks (*Cairina moschata*, *Anas platyrhynchos*, *Dendrocyga autumnalis*)

In the black bellied whistling duck (*Dendrocyga autumnalis*) in Southern Texas a wide range of parasites were identified in the gastrointestinal tract. *Echinuria uncinata*, *Tropisursus crami*, *Parhadjelia neglecta*, *Anomotaenia* spp., *Apatemon gracilis*, *Zygocotyle lunata*, *Porrocaecum* spp., *Corynosoma* spp., *Corynosoma peposacae*, *Dicranotaenia* spp., *Echinostoma revolutum*, *Sobolevicanthus* spp. and *Cloacataenia megalops* were identified but seemed to have no effect on the ducks’ physical condition [69]. In Egypt different breeds of ducks were analysed for gastrointestinal parasites. *Ascaridia galli*, *Heterakis gallinarum*, *Cladogynia phoeniconaiadis*, *Echinolepis carioca*, *Baerfainia anoplocephaloides*, *Echinoparyphium paraulum*, *Echinoparyphium recurvatum* and *Entamoeba gallinarum* were found in a wide range of ducks [70].

In Tanzania, parasites of free range adult ducks were identified. The parasites reported to have been identified were *Ascaridia coluba*, *Ascaridia dissimilis*, *Ascaridia galli*, *Capillaria anatis*, *Capillaria annulata*, *Capillaria contorta*, *Heterakis dispar*, *Heterakis gallinarum*, *Raillietina echinoborthrida*, *Raillietina tetragona*, *Subulura brumpti*, *Subulura sucturia* [71]. The author stated potentially pathogenic worms such as *Ascaridia galli* and *Heterakis gallinarum* had no clear physical effect on the health status of the ducks [70]. Similar work was done in free ranging ducks in Kenya. Parasites found and included; *Gongylonema ingluvicola*, *Heterakis gallinarum*, *H. isolonche*, *Capillaria contorta*, *Sublura brumpti*, *Ascaridia galli* and *Hymenolepis* spp. [72].

In Eastern Kenya, muscovy ducks at different locations were slaughtered and the presence of endoparasites investigated. The investigation found the following; *Ascaridia galli*, *Trichostrongylus tenus*, *Heterakis gallinarum*, *Sublura brumpti*, *Capillaria contorta*, *C. annulata*, *Tetrameres fissipina*, *Raillietina echinoborthida*, *R. tetragona* and *Hymenolepis cantaniana* [73]. In Southwestern Nigeria gastrointestinal parasites that inhabited the domestic duck (*Anas platrhynchos*) was studied. The study revealed the following parasites; *Ascaridia galli*, *Heterakis gallinarum*, *Capillaria* spp., *Syngamus trachea*, *Echinuris uncinata*, *Tyzzeria* spp., *Eimeria* spp. and *Cryptosporidium* spp. Although numerous parasites were found no quantification was made and no information was presented on the body condition of the animal pre- or post mortem [74].

In central Mexico and the south-western United States twenty-five species of helminths were recovered from the gastrointestinal trach of the wild Mexican duck (*Anas platyrhynchos diazi*) [75]. The parasites recorded were; *Echinopartphium recurvatum*, *Echinostoma revolutum*, *Hypoderaeum conoideum*, *Notocotylus attenuatus*, *Prosthogonimus cuneatus*, *Zygocotyle lunata*, *Anomotaenia ciliate*, *Cloacataenia megalops*, *Diorchis bulbodes*, *Diorchis* spp., *Drepanidotaenia lanceolata*, *Echinocotyle rosseteri*, *Fimbriaria fasciolaris*, *Fimbriaroides* spp., *Hymenolepis* spp. (1), *Hymenolepis* spp. (2), *Sobolevicanthus gracilis*, *Corynosoma constrictum*, *Polymorphus minutus*, *Amidostomum acutum*, *Echinuria* spp., *Epomidostomum crami*, *Hystrichis varispinosus*, *Tetrameres* spp. and *Rusguniella arctica* [76]. A similar study on wild ducks (*Anas platyrhynchos diazi*) in Mexico was done for identification of helminths. Eight species of helminths were identified and included; *Zygocotyle lunata*, *Echinostoma revolutum*, *Psilostromum ondatrae*, *Amidostomum anseris*, *Capillaria* spp., *Corynosoma* spp., *Polymorphus boschadis* and *Hymenolepis megalops*. However, no data was provided on the body condition or the live-weight of the ducks which harbored these parasites [76].

In the Czech Republic, the mallard duck (*Anas platyrhynchos*) is reared on breeding farms were sampled for gastrointestinal parasites. The study found a low prevalence of gastrointestinal parasites in ducks and coccidia and *Amidostomum anseris* were the only parasites found [77]. In Bangladesh, the domestic duck (*Anas platyrhynchos*) and the Muscovy duck (*Cairina moschata*) were evaluated for gastrointestinal parasites. The domestic duck had *Ascaridia* spp., *Heterakis* spp., *Capillaria* spp. and *Eimeria* spp. The Muscovy duck had only *Eimeria* spp. and *Ascaridia* spp. [78]. Similar work was done in Japan with anseriforms which lived in the wild. The six parasites found in ducks (*Anas platyrhynchos*) and included; *Tetrameres fissipina*, *Streptocara crassicauda*, *Epomidostomum crami*, *Amidostomum anseris*, *Capillaria anatis* and *Eocoleus contortus* but no correlation was made between the presence of the parasites and the body condition of the ducks [79]. Seven parasites were reported to have been found in the gastrointestinal tracts of mallard duck (*Anas platyrhynchos*). The parasites involved were as followed; *Echinostoma revolutum*, *Hymenolepis* spp., *Cotylurus formis*, *Tetrameres* spp., *Zygocotyle* spp., *Amphirus elongatus* and *Filicollis* spp. but no records of the animals’ body condition or weights were given [80].

The effects of *Histomonas meleagridis* on the performance of Muscovy ducks (*Cairina moschata*) and mule ducks (F_1_ *Cairina moschata* and *Anas platyrhynchos*) was done experimentally. Authors reported that there was no difference in weight gain, mortality and clinical lesion between ducks infected and the control over at thirty-five-day period. These results showed that the ducks were resistant to clinical disease but can be used as carriers for the disease to susceptible species [81]. In Italy an outbreak of *Streptocara incognita* was reported in Muscovy ducks (*Carina moschata domesticus*). The infected ducks were in poor body condition with pectoral muscle atrophy. *Streptocara incognita* was found in the oesophagus and caused dilation and discrete diphtheric lesion underneath a pseudo membrane. Post-mortem examination revealed pseudo membrane oesophagitis which occluded the lumen of the oesophagus [82].

*Cryptosporidium* spp. has been found in farmed ducks in Germany. The reports showed that there were no correlations between the presence of *Cryptosporidium* and poor performance, clinical signs of ill health or gastrointestinal lesion [83] (Table 6). *Cochlosoma rostratum* can be found in many domestic ducks (*Anas platyrhynchos* and *Carina moschata*) and was observed in sick and healthy birds. No difference was noted in the faeces and intestinal contents of parasitized and non-parasitized birds. Sick birds were cases of bacterial infection with heavy infection of *Cochlosoma rostratum* but inflammation of the digestive tract was absent [84]. Ducklings were experimentally infected with *Coclosoma anatis* to observe the effect of the parasite on the birds’ performance. Results reported showed infection with *Cochlosoma anatis* did result in lower palatinase and maltase activity in the small intestine but Sucrase activity was significantly increased. However, there was no overt clinical sign of gastrointestinal illness or weight loss observed [85]. Ducklings (*Cairina moschata*) were inoculated with *Cochlosoma anatis* had lower mean body weights than the control group. Clinical observations and parasitological findings correlated *Cochlosoma anatis* as a possible cause for runting syndrome in ducklings [86]. The efficacy of Toltrazuril on *Eimeria mulardi* in mule ducks (F_1_ *Anas platyrhynchos* and *Cairina moschata*) was investigated. Authors noted that treatment of infected ducks five days’ post-inoculation received similar results as ducks (healthy) that were not exposed to *Eimeria mulardi* [32] (Table 2).

## 8. Conclusions

Generally, neo-tropical animals in the wild were observed as reservoirs of the parasites. Several authors paid little to any attention to the effect these parasites have on the animals’ body condition and body weight. South American Camelids, ducks, chinchillas, turkeys and guinea pigs was found to have had numerous parasites present in their gastrointestinal tracts. However, these were only a few cases where these were observed to have been experiencing ill health due to gastrointestinal parasites. The animals in this review were found free ranging or in the wild and seemed to have the ability to harbour these parasites without any overt clinical effect. This can pose health risks to humans and introduce these parasites to domesticated livestock species. Further studies should be conducted to observe the effect gastrointestinal parasites have on production and health on Neo-tropical Domesticated animals (South American Camelids, ducks, chinchillas, turkeys and guinea pigs). The majority of the literature reviewed was reported in the last twenty years. This sheds light on the improved diagnostic technology as well as the recent interest in the endoparasites of these animals present in the wild.

## Figures and Tables

**Table 1:** Gastrointestinal Parasites present in South American Camelids and its effect on the animals.

Parasites	Location	Pathological Lesions or Clinical Effects	Confirmatory Test	Year	Ref.
*Bunostomum*, *Camelostrongylus*, *Capillaria*, *Trichostrongylus*, *Cooperia*, *Nematodirus*, *Ostertagia*, *Marshallagia Trichuris* spp.	England and Wales	enlarged lymph nodes, subcutaneous oedema, ascites, hydrothorax pulmonary oedema	Necropsy	2014	[20]
Strongyles, *Nematodirus* spp., *Giardia* spp.	Wisconsin	unformed stool poor condition	Coproscopy	1987	[21]
*Camelostrongylus mentalatus Trichostrongylus* spp., *Nematodirus*, *Trichuris*, *Eimeria macusaniensis Eimeria* spp.	Britain	diarrhoea, weight loss and death	Necropsy	2008	[22]
*Nematodirus* spp., *Eimeria macusaniensis*, Strongylida	Chile	Not recorded	Coproscopy	2012	[23]

**Table 2:** Drugs used in the Treatment of endoparasites in Non-Domesticated Neo-Tropical Animals.

Host	Drug	Parasite	Ref.
South American Camalids (Lamas)	Fenbendazole (5 mg/kg)	*Trichuris* spp., *Strongyloides* spp., *Nematodirus* spp.	[27]
Lamas and alpacas	Ivermectin (0.2 mg/kg)	*Oesophagostomum* spp., *Trichostrongylus* spp.	[28]
Lamas	Decoquinate (0.5 mg/kg) Amprolium (5 mg/kg)	Coccidia	[19,24]
Guinea pigs	Sulphaquinalones	*Eimeria caviae*	[30]
Guinea pigs	Piperazine	*Paraspidodera uncinata*	[31]
Mule ducks (F_1_ *Anas platyrhynchos* and *Cairina moschata*)	Toltrazuril	*Eimeria mulardi*	[32]

**Table 3:** Gastrointestinal Parasites present in Guinea pigs and its effect on the animals.

Parasites	Location	Pathological Lesions or Clinical Signs	Confirmatory Test	Year	Ref.
*Capillaria hepatica*, *Graphidoides mazzai*, *Trichuris gracilis*, *Paraspidodera uncinata*, *Fasciola hepatica*	Chile	Not Recorded	Necropsy	2002	[34]
*Paraspidodera uncinata*	Brazil	Not recorded	Necropsy	2002	[35]
*Nippostrongylus* spp., *Paraspidodera uncinata* *Eimeria caviae*	Italy	Not recorded	Coproscopy	2015	[36]
*Paraspidodera uncinata*, *Balantidium coli*	Iran	Not Recorded	Coproscopy	2014	[38]
*Graphydium strigosum*, *Trichostrongylus* spp., *Paraspidodera uncinata*, *Eimeria caviae*	Congo	Not Recorded	Coproscopy	2015	[41]

**Table 4:** Gastrointestinal Parasites present in Chinchillas and its effect on the animals.

Parasites	Location	Pathological Lesions or Clinical Signs	Confirmatory Tests	Year	Ref.
*Giardia* Assemblage A, B	Europe	Not Recorded	Coproantigen analysis	2014	[39]
*Giardia* Assemblage B, C	Italy	Not Recorded	Direct Fluorescent Assays and Genetic Sequencing	2012	[44]
*Hymenolepis* spp.	Italy	Not Recorded	Coproscopy	2015	[36]
*Giardia duodenalis* Assemblage B, C, A, E	Belgium	Not Recorded	Coproantigen Analysis	2011	[49]
*Giardia duodenalis* Assemblage B	Brazil	No clinical signs for gastrointestinal illness was observed	Coproantigen analysis	2011	[50]
*Giardia* spp., *Eimeria* spp., *Trichostrongylus* spp., *Trichuris* spp., *Cryptosporidium* spp., *Saccharomyces glutauratus*	Argentina	No illness or deaths were observed	Necropsy	2017	[51]

**Table 5:** Gastrointestinal Parasites present in Turkeys and its effect on the animals.

Parasites	Location	Pathological Lesions or Clinical Signs	Confirmatory Test	Year	Ref.
*Davainea ransomi*, *Davainea fuhrinanni*, *Metroliasthes lucida*	Philadelphia	Not Recorded	Necropsy	1931	[53]
*Eimeria meleagridis*, *Histomonas meleagridis*, *Trichomonas* spp.	Philadelphia	Not Recorded	Coproscopy	1948	[54]
*Echinoparyphium recurvatum*, *Zygocotyle lunata*, *Stomylotrema vivarium*, *Strigea elagans meleagris*, *Prostogonimus ovatus*, *Echinostoma revolutum*, *Ascocotyle* spp., *Brachylaima virginianum*, *Tanaisia* spp., *Zonorchis* spp., *Metroliasthes lucida*, *Raillietina geogiensis*, *Raillietina ransomi*, *Raillietina cesticillus*, *Davainea meleagridis*, *Hymenolepis carioca*, *Strongyloides* spp., *Trichostrongylus tenuis*, *Dispharynx nasuta*, *Cyrnea eurycerca*, *Ascaridia dissimilis*, *Capillaria* spp., *Cyrnea* spp., *Singhfilaria hayesi*, *Heterakis gallinarum*, *Aproctella stoddardi*, *Synlimantus* spp., *Aulonocephalus pennula*, *Capillaria* spp., *Chandlerella* spp., *Cheilospirura spinose*, *Splendidofliaria* spp., Slendidofilariinae. *Mediohynchus papillosum*	Florida	Not Recorded	Necropsy	1975	[55]
*Brachylaema virginiana*, *Cotylurus flabelliformis*, *Echinoparyphium recurvatum*, *Zygocotyle lunata*. *Davainea meleagradis*, *Hymenolepis cantaniana*, *Hymenolepis carioca*, *Hymenolepis* spp., *Metroliasthes lucida*, *Raillietina georgiensis*, *Raillietina williamsi*, *Raillietina ransomi*, *Ascaridia dissimilis*, *Ascaridia galli*, *Capillaria annulata*, *Capillaria bursata*, *Capillaria obsignata*, *Dispharynx nasuta*, *Gongylonema ingluvicola*, *Heterakis gallinae*, *Seurocyrnea colini*, *Seurocyrnea* spp., *Strongyloides avium*, *Strongyloides* spp., *Trichostrongylus tenuis*	United States	Not Recorded	Necropsy	1963	[56]
*Hymenolepis carioca*, *Metroliasthes lucida*, *Raillietina georgensis*, *Raillietina williamsi*, *Ascaridia dissimilis*, *Capillaria caudinflata*, *Heterakis gallinarum*	Kentucky Tennessee	Not Recorded	Necropsy	1984	[57]
*Eimeria meleagrimitis*, *Eimeria gallopavonis*, *Eimeria meleagridis*, *Eimeria dispersa*, *Eimeria innocua-Eimeria subrontunda*	United States	Not Recorded	Coproscopy	1988	[58]
*Ascaridia dissimilis*, *Heterakis gallinarum*, *Eimeria dispersa*, *Raillietina* spp.	Arkansas	Not Recorded	Coproscopy	1990	[59]
*Echinoparyphium recurvatum*, *Echinostoma revolutum*, *Metroliasthes lucida*, *Imparmargo baileyi*, *Railletiena* spp., *Choanataenia* spp., *Mediorhynchus grandis*, *Heterakis gallinarum*	Kansas	Not Recorded	Necropsy	2003	[60]
*Cheilospirura hamulosa*, *Heterakis gallinarum*, *Baruscapillaria obsignata*, *Eucoleus annulatus*, *Hymenolepis cantaniana* and *Raillietina tetragona*	Brazil	Chronic diffuse inflammation with granulocyte infiltration (2.2–8.8 kg LW)	Necropsy	2006–2008	[61,62,63,64]
*Choanotaenia infundibulum*, *Davainea meleagridis*, *Echinolepsis carioca*, *Fimbriaria fasciolaris*, *Microsomacanthus paracompressa*, *Raillietina circumvallata*, *Raillietina cestillus*, *Raillietina echinoborthida*, *Raillietina ransomi*, *Raillietina tetragona*, *Raillietina williamsi* and *Sobolevicanthus gracilis*, *Ascaridia dissimilis*, *Ascaridia galli*, *Capillaria bursata*, *Capillaria caudinflata*, *Capillaria annulatus*, *Eucoleus annulatus*, *Ganguleterakis dispar*, *Heterakis gallinarum*, *Syngamus trachea*, *Thominx phasiania*, *Trichostrongylus tenuis*, *Echinoparyphium recurvatum*, *Echinoparypium rubrum*, *Echinostoma revolutum*, *Echinostoma trivolis*, *Notocotylus attenuatus*, *Prosthogonimus cuneatus Prostogonimus ovatus*, *Mediorynchus* spp.	Nebraska	Not Recorded	Necropsy	2005	[65]
*Cryptosporidium* spp.	Missouri	Huddling, diarrhoea, villous atrophy, cryptic hypertophy	Coproscopy	1988	[66]
*Cryptosporidium* spp.	Iowa	No clinical illness or lesions	Coproscopy	1988	[67]

**Table 6:** Gastrointestinal Parasites present in ducks and its effect on the animals.

Parasites	Location	Pathological Lesions or Clinical Signs	Confirmatory Tests	Year	Ref.
*Echinuria uncinata*, *Tropisursus crami*, *Parhadjelia neglecta*, *Anomotaenia* spp., *Apatemon gracilis*, *Zygocotyle lunata*, *Porrocaecum* spp., *Corynosoma* spp., *Corynosoma peposacae*, *Dicranotaenia* spp., *Echinostoma revolutum*, *Sobolevicanthus* spp., *Cloacataenia megalops*	Texas	No effect on duck’s physical condition	Necropsy	1975	[69]
*Ascaridia galli*, *Heterakis gallinarum*, *Cladogynia phoeniconaiadis*, *Echinolepis carioca*, *Baerfainia anoplocephaloides*, *Echinoparyphium paraulum*, *Echinoparyphium recurvatum*, *Entamoeba gallinarum*	Egypt	Not Recorded	Necropsy	2011	[70]
	Tanzania	No clear physical effect on the health	Necropsy	2007	[71]
*Gongylonema ingluvicola*, *Heterakis gallinarum*, *H. isolonche*, *Capillariacontorta*, *Sublura brumpti*, *Ascaridia galli*, *Hymenolepis* spp.	Kenya	Not Recorded	Necropsy	2018	[72]
*Ascaridia galli*, *Trichostrongylus tenus*, *Hetrerakis gallinarum*, *Sublura brumpti*, *Capillaria contorta*, *C.annulata*, *Tetrameres fissipina*, *Raillietina echinoborthida*, *R. tetragona*, *Hymenolepsis cantaniana*	Kenya	Not Recorded	Coproscopy	2015	[73]
*Ascaridia galli*, *Heterakis gallinarum*, *Capillaria* spp., *Syngamus trachea*, *Echinuris uncinata*, *Tyzzeria* spp., *Eimeria* spp., *Cryptosporidium* spp.	Nigeria	Not Recorded	Coproscopy	2011	[74]
*Echinopartphium recurvatum*, *Echinostoma revolutum*, *Hypoderaeum conoideum*, *Notocotylus attenuatus*, *Prosthogonimus cuneatus*, *Zygocotyle lunata*, *Anomotaenia ciliate*, *Cloacataenia megalops*, *Diorchis bulbodes*, *Diorchis* spp., *Drepanidotaenia lanceolata*, *Echinocotyle rosseteri*, *Fimbriaria fasciolaris*, *Fimbriaroides* spp., *Hymenolepis* spp., *Sobolevicanthus gracilis*, *Corynosoma constrictum*, *Polymorphus minutus*, *Amidostomum acutum*, *Echinuria* spp., *Epomidostomum crami*, *Hystrichis varispinosus*, *Tetrameres* spp., *Rusguniella arctica*	Mexico and USA	Not Recorded	Necropsy	1986	[75]
*Zygocotyle lunata*, *Echinostoma revolutum*, *Psilostromum ondatrae*, *Amidostomum anseris*, *Capillaria* spp., *Corynosoma* spp., *Polymorphus boschadis*, *Hymenolepis megalops*	Mexico	Not Recorded	Necropsy	2010	[76]
*Ascaridia* spp., *Heterakis* spp., *Capillaria* spp., *Eimeria* spp.	Bangladesh	Not Recorded	Coproscopy	2014	[77]
*Tetrameres fissipina*, *Streptocara crassicauda*, *Epomidostomum crami*, *Amidostomum anseris*, *Capillaria anatis*, *Eocoleus contortus*	Japan	Not Recorded	Necropsy	2009	[79]
*Echinostoma revolutum*, *Hymenolepis* spp., *Cotylurus formis*, *Tetrameres* spp., *Zygocotyle* spp., *Amphirus elongates*, *Filicollis* spp.	Michigan	Not Recorded	Coproscopy	1938	[80]
*Streptocara incognita*	Italy	Poor body condition	Copsroscopy	2005	[82]
*Cryptosporidium* spp.	Germany	No signs of ill health	Coproscopy	1994	[83]
